# “Will I change nodule management recommendations if I change my CAD system?”—impact of volumetric deviation between different CAD systems on lesion management

**DOI:** 10.1007/s00330-023-09525-z

**Published:** 2023-03-10

**Authors:** Alan A. Peters, Andreas Christe, Oyunbileg von Stackelberg, Moritz Pohl, Hans-Ulrich Kauczor, Claus Peter Heußel, Mark O. Wielpütz, Lukas Ebner

**Affiliations:** 1 Department of Diagnostic and Interventional Radiology, University Hospital of Heidelberg, Im Neuenheimer Feld 672, Heidelberg, Germany; 2 Translational Lung Research Center Heidelberg (TLRC), German Lung Research Center (DZL), Marsilius-Arkaden 130, 69120 Heidelberg, Germany; 3 Department of Diagnostic and Interventional Radiology With Nuclear Medicine, University Hospital of Heidelberg, Thoraxklinik Heidelberg, Roentgenstrasse 1, 69126 Heidelberg, Germany; 4 Department of Diagnostic, Interventional and Pediatric Radiology, Inselspital, Bern University Hospital, University of Bern, 3010 Freiburgstrasse, Switzerland; 5 Institute of Medical Biometry, University of Heidelberg, Im Neuenheimer Feld 130.3, 69120 Heidelberg, Germany

**Keywords:** Lung neoplasms, Artificial intelligence, Deep learning, Computer-assisted diagnosis, Imaging phantoms

## Abstract

**Objectives:**

To evaluate and compare the measurement accuracy of two different computer-aided diagnosis (CAD) systems regarding artificial pulmonary nodules and assess the clinical impact of volumetric inaccuracies in a phantom study.

**Methods:**

In this phantom study, 59 different phantom arrangements with 326 artificial nodules (178 solid, 148 ground-glass) were scanned at 80 kV, 100 kV, and 120 kV. Four different nodule diameters were used: 5 mm, 8 mm, 10 mm, and 12 mm. Scans were analyzed by a deep-learning (DL)–based CAD and a standard CAD system. Relative volumetric errors (RVE) of each system vs. ground truth and the relative volume difference (RVD) DL–based vs. standard CAD were calculated. The Bland–Altman method was used to define the limits of agreement (LOA). The hypothetical impact on LungRADS classification was assessed for both systems.

**Results:**

There was no difference between the three voltage groups regarding nodule volumetry. Regarding the solid nodules, the RVE of the 5-mm-, 8-mm-, 10-mm-, and 12-mm-size groups for the DL CAD/standard CAD were 12.2/2.8%, 1.3/ − 2.8%, − 3.6/1.5%, and − 12.2/ − 0.3%, respectively. The corresponding values for the ground-glass nodules (GGN) were 25.6%/81.0%, 9.0%/28.0%, 7.6/20.6%, and 6.8/21.2%. The mean RVD for solid nodules/GGN was 1.3/ − 15.2%. Regarding the LungRADS classification, 88.5% and 79.8% of all solid nodules were correctly assigned by the DL CAD and the standard CAD, respectively. 14.9% of the nodules were assigned differently between the systems.

**Conclusions:**

Patient management may be affected by the volumetric inaccuracy of the CAD systems and hence demands supervision and/or manual correction by a radiologist.

**Key Points:**

• *The DL-based CAD system was more accurate in the volumetry of GGN and less accurate regarding solid nodules than the standard CAD system.*

• *Nodule size and attenuation have an effect on the measurement accuracy of both systems; tube voltage has no effect on measurement accuracy.*

• *Measurement inaccuracies of CAD systems can have an impact on patient management, which demands supervision by radiologists.*

## Introduction

The widespread routine clinical implementation of lung cancer screening programs all over the world is directly linked to the increasing importance of standardized pulmonary nodule management. Current clinical practice guidelines rely on precise and reproducible measurements of the detected pulmonary nodules. Since different definitions of the nodule diameter can be applied (e.g., mean or maximum diameter), volumetry of pulmonary nodules is deemed the most accurate predictor of lung cancer risk [1, 2] and may account for irregular shapes, air spaces, and mixed solid and subsolid compartments [3]. Furthermore, the repeatability of diameter measurements is known to be suboptimal [4]. Therefore, patient management proposed in the latest guidelines by the British Thoracic Society (BTS), the Nederlands-Leuvens Longkaker Screening Onderzoek (NELSON) group, or the American College of Radiology (ACR) is mainly based on nodule volumetry [5–7].

In front of an increasing number of screening examinations and the associated additional workload, careful CT reading and precise measurements are challenging for radiologists in daily clinical routine. A nodule measurement variability of ± 25% has been demonstrated in several in vivo “coffee-break” studies, in which individuals were scanned twice on the same day [8, 9]. A postulated solution to tackle the increasing workload while at the same time maintaining or even improving nodule measurement accuracy is the introduction of artificial intelligence (AI)–based computer-aided diagnosis (CAD) systems into clinical routine. Further advantages of such systems comprise the automation of repetitive radiological duties and the avoidance of reader fatigue, which have been recognized as an increasing focus in the field [10]. In the context of pulmonary nodule detection, deep learning (DL)–based CAD systems have proven to be helpful as first or second reader devices [11–13]. Since it is very likely that the majority of pulmonary nodule analyses will be performed by software and not by human readers in the near future, it seems pertinent to evaluate the performance of DL-based CAD systems in this regard. It is tempting to speculate whether novel DL-based CAD systems will have similar or lower inter-reader variability than different human individuals.

The influence of scanning parameters and reconstruction on nodule volumetry is well appreciated [14, 15]. In addition, there are studies comparing different software solutions in the chest area focusing on functional lung parameters [16]. But only few studies have compared different software packages regarding the accuracy of pulmonary nodule volumetry [17, 18].

The primary aims of this study were to evaluate the measurement accuracy of a commercially available DL-based CAD system and a standard CAD system with an in-built semi-automatic volumetry tool in comparison to the true volume of artificial pulmonary nodules and to evaluate the impact on patient management recommendations. The secondary aim was to analyze the influence of tube voltage, nodule size, and attenuation on the measurement accuracy of the CAD systems.

## Materials and methods

### Chest phantom

A previously described dedicated anthropomorphic chest phantom equipped with artificial nodules was utilized in this study (Lungman Phantom; Kyoto Kagaku Co., Ltd.) [12, 19].

Nodules of four different diameters (corresponding volumes) were used: 5 mm (65.5 mm^3^), 8 mm (268.1 mm^3^), 10 mm (523.1 mm^3^), and 12 mm (904.8 mm^3^). Two density types of nodules were used, nodules with a density of + 100 Hounsfield units (HU) to simulate solid lesions and nodules with a density of − 630 HU to simulate ground-glass nodules (GGN).

**Fig. 1:**
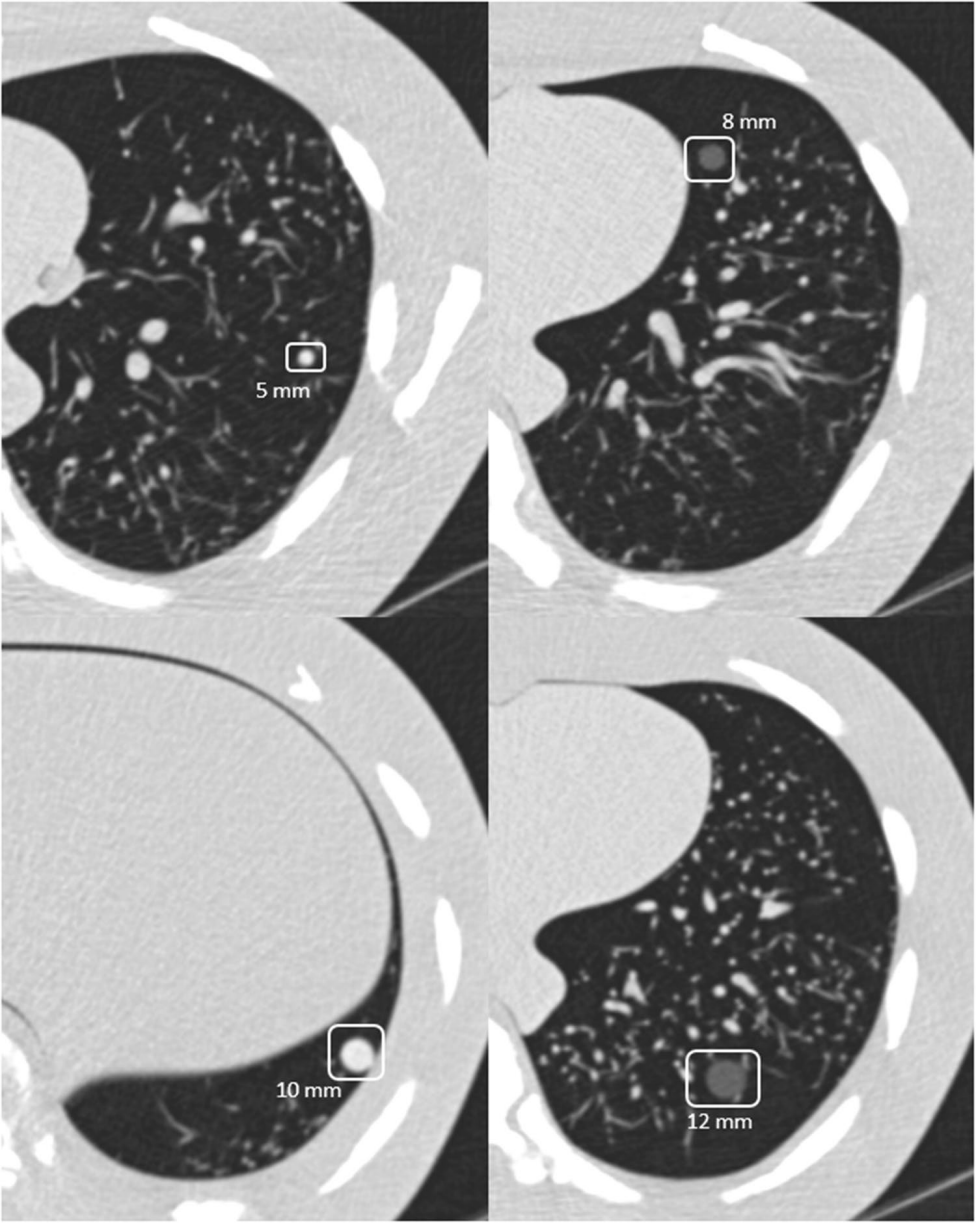
Examples of the utilized solid and ground-glass nodules of the four size groups (window center/width − 500/1500 HU)

In total, 59 different phantom arrangements with 326 artificial nodules (178 solid, 148 ground-glass) were analyzed. Each phantom was equipped with zero to eight nodules. A random generator decided the nodule distribution (number, side, type, size, lung segment, and peripheral versus central location) within the simulated lung parenchyma. Exemplary nodules are depicted in Fig. 1.

### Chest computed tomography scan parameters

All examinations were performed on a 64-row multidetector CT scanner (Somatom Sensation 64, Siemens) with the following scan parameters: spiral acquisition mode, 24 × 1.2 mm, pitch, 0.8; slice thickness, 1.5 mm, increment, 1.5 mm, field of view, 35 cm, and scan length, 33 cm. For image reconstruction, filtered back projection (FBP) was utilized with kernels of B30f (soft kernel) and B70f (hard kernel) and lung window setting (center, − 500 HU; width, 1500 HU). Each phantom arrangement was scanned with tube voltages of 80, 100, and 120 kV. Automated modulation of the tube current (CareDose4D) was used to maintain independence from body weight and body mass index (BMI) and to approximate routine scans. The mean volume computed tomography dose indices (CTDIvol) of the three voltage groups were 1.2 ± 0.6 mGy, 2.1 ± 1.0 mGy, and 3.1 ± 1.4 mGy; the corresponding mean effective doses (*E*) were 0.9 mSv, 1.6 mSv, and 2.3 mSv. The chest phantom CT data sets were used in an earlier study already [20].

### CAD systems

A commercially available deep-learning (DL)–CAD system was used in this study (InferRead CT Lung, Infervision Medical Technology Co., Ltd.). The deep-learning model was principally built with two convolutional neural network (CNN) models: a DenseNet model (feature map extractor) and a Faster R-CNN–based model (detector).

Since the CT scans have a property of 3D image volume, the Faster R-CNN network of this system was modified to take consecutive sections as input and to form a multichannel “2.5D CNN.” The dimension “2.5D” implies that the model is not exact 3D convoluted due to the segmented axial data and their heterogeneous resolutions.

In this study, instead of regular CNN, the DenseNet model was used to extract the features and back propagate them. In contrast to regular CNN, where feature maps are usually connected at one go, feature maps in DenseNet are directly connected one by one, thus forming a densely connected network with a smaller number of layers. The feature density can thus be maintained during the propagation process, and the model will possess a higher overall expressive power.

The standard CAD system used is an established, commercially available software (Syngo.CT Lung CAD, Syngo.Via client 6.4, Siemens Medical Solutions) designed to work as a second-reader tool. It automatically pre-processes and marks nodules and measures them semi-automatically offering the possibility of subsequent manual modification. An integrated software tool allowed dedicated analysis of subsolid and solid lesions.

### Statistical analysis

The average volume uses all measured volumes and is a first descriptive measure for the software outputs. A more detailed measure is the relative volumetric error (RVE). The RVE is calculated for each nodule and accounts for the known volume as ground truth (GT). It was calculated by using the following formula:

$$\mathrm{RVE}=\frac{\mathrm{Measured}\;\mathrm{volume}-\mathrm{GT}}{\mathrm{GT}}$$

The RVE for the different voltage and size groups was compared with descriptive measures (mean, standard deviation) and additionally with the Friedman test for paired samples or, alternatively, the Kruskal–Wallis test for unpaired samples.

The absolute and relative volume differences (AVD, RVD) between the systems were calculated by using the following formulas:

$$\mathrm{AVD}=V_{\mathrm{AICAD}}-V_{\mathrm{standard}\;\mathrm{CAD}}$$

$$\mathrm{RVD}=\frac{\mathrm{AVD}}{V_{\mathrm{standard}\;\mathrm{CAD}}}$$

The Bland–Altman method with limits of agreement (LOA) was utilized to assess the variability between the volumetric measurements of the systems. In line with the literature, the RVD of each measured nodule volume was plotted against the respective average volume [8, 21–23]. The upper and lower LOA were calculated as the range of 95% of the observed relative differences in the two volume measurements (1.96 standard deviations above and below the mean difference). The hypothetical LungRADS categories were compared with the Wilcoxon test between the two systems; the systems’ rates of correctly classified nodules were compared by using the McNemar test. Data analysis was performed on a dedicated statistic software (SPSS Statistics, IBM Corp., version 25.0). Since this was an exploratory and descriptive analysis, all *p* values were interpreted in a descriptive manner, with *p* < 0.05 indicating statistical significance.

## Results

The DL-based CAD software automatically detected 97.9% of all nodules (*n* = 319/326). The missed nodules (four solid, three GGN) could not be added to the analysis manually as this function was not implemented in the utilized version of the software. Regarding the distribution of the missed nodules, two were located in the upper lobes, three in the middle lobe/lingual, and two in the lower lobes. Regarding their size groups, there was one 5-mm nodule and two nodules from each of the remaining size groups (8 mm, 10 mm, and 12 mm) missed.

The standard CAD system detected 81.6% (*n* = 266/326) of the nodules automatically, but the missed nodules (54 GGN, 6 solid) could be manually added to the analysis.

### Tube voltage has no effect on nodule volumetry

**Fig. 2:**
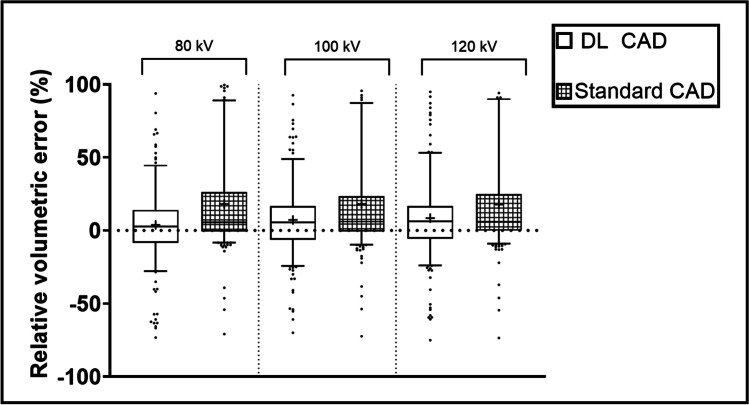
Boxplots showing the RVE of all nodules by tube voltage group depicted as 5^th^–95^th^ percentile. RVE = relative volumetric error

The detection rates of the DL-based CAD system per voltage group were 94.2% (*n* = 307/326), 97.2% (*n* = 317/326), and 97.9% (*n* = 319/326) for 80 kV, 100 kV, and 120 kV, respectively. The mean RVEs of the different voltage groups for DL CAD/standard CAD regarding solid nodules were 1.9/0.2%, 0.2/ − 0.02%, and 1.3/1.0% for 80 kV, 100 kV, and 120 kV, respectively (Fig. 2). There were no significant differences between the three voltage groups (80 kV vs. 100 kV vs. 120 kV) regarding solid nodules, neither with the DL-based CAD (*p* = 0.193) nor with the standard CAD system (*p* = 0.135).

The corresponding RVEs for DL CAD/standard CAD regarding GGN were 6.1/39.7%, 15.8/40.0%, and 17.0/38.0%.

A subgroup analysis indicated a difference in RVE between the 80- and the 120-kV group in the volumetry of the DL-based CAD system (*p* = 0.008). However, there was no difference observed between the voltage groups among the GGN with the standard CAD system (*p* = 0.192).

### Nodule attenuation and size have an effect on volumetry

Regarding the different size groups of the solid nodules, the RVE of the DL-based CAD system was lower for the 8-mm and 10-mm nodules compared to the 5-mm and 12-mm nodules. The corresponding results for the standard CAD system showed that the 5-mm and 8-mm nodules had higher RVEs compared to the 10-mm and 12-mm nodules.

Regarding the GGN, both systems had higher volumetric errors and higher standard deviations compared to the solid nodules; the DL-based CAD showed lower mean RVEs compared to the standard CAD. Both systems had higher RVEs in measuring the 5-mm GGN compared to the other size groups.

**Fig. 3:**
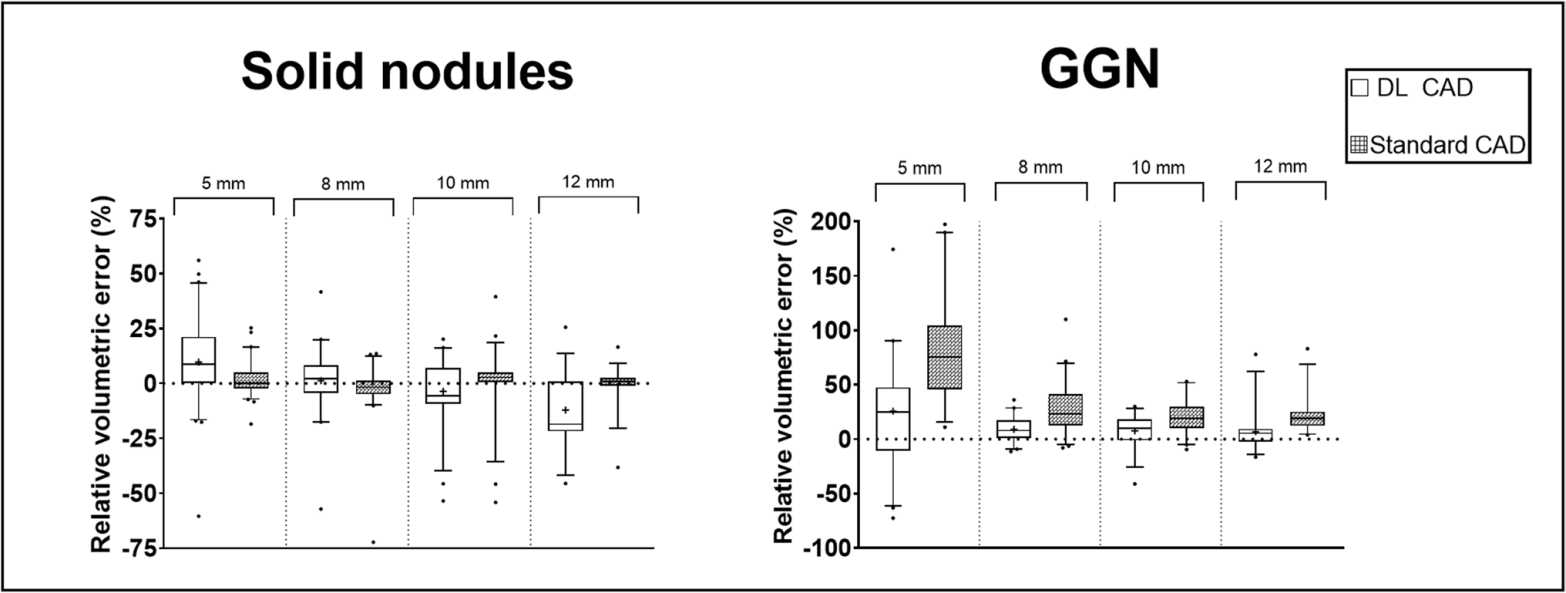
Boxplots showing the RVE by size group for solid nodules and for GGN depicted as 5^th^ to 95^th^ percentile. GGN = ground-glass nodules, RVE = relative volumetric error

The RVEs of all groups are shown in Table 1 and depicted as boxplots in Fig. 3.

**Table 1:** RVE by size group and density

Nodule density	Size group (volume)	DL-based CAD Mean RVE (SD), %	Standard CAD Mean RVE (SD), %
Solid	5 mm (65.5 mm^3^)	12.2 (20.8)	2.8 (8.3)
	8 mm (268.1 mm^3^)	1.3 (13.5)	− 2.8 (11.5)
	10 mm (523.6 mm^3^)	− 3.6 (14.0)	1.5 (13.3)
	12 mm (904.8 mm^3^)	− 12.1 (15.3)	− 0.3 (8.0)
GGN	5 mm (65.5 mm^3^)	25.6 (44.8)	81.0 (47.6)
	8 mm (268.1 mm^3^)	9.0 (10.7)	28.0 (22.8)
	10 mm (523.6 mm^3^)	7.6 (14.3)	20.6 (15.0)
	12 mm (904.8 mm^3^)	6.8 (17.2)	21.2 (15.5)

*CAD* computer-aided diagnosis, *DL* deep learning, *GGN* ground-glass nodule, *RVE* relative volumetric error, *SD* standard deviation

### Software comparison

**Fig. 4:**
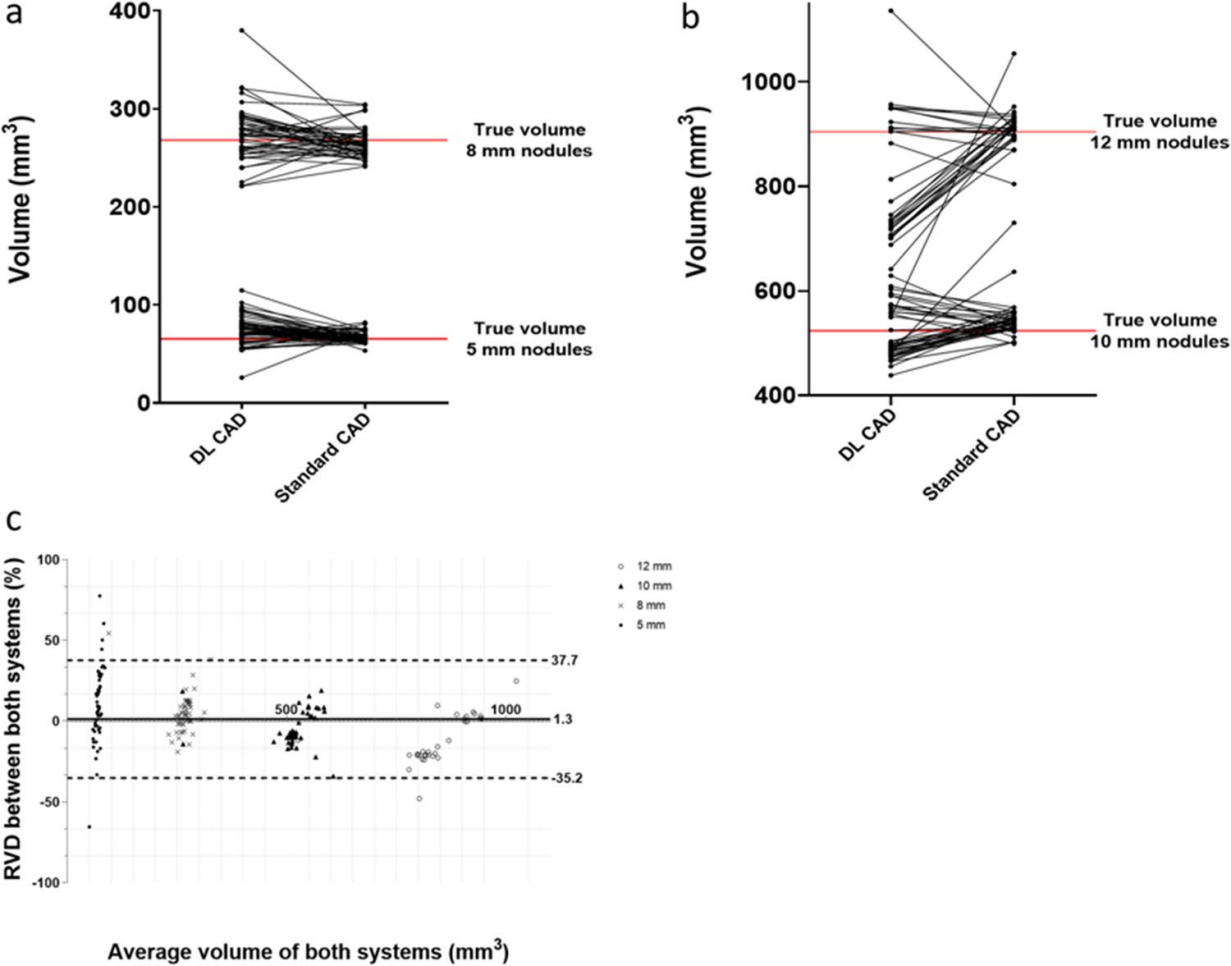
Software comparison depicted as spaghetti plots showing the dependent measurements of (**a**) 5-mm and 8-mm nodules and (**b**) 10-mm and 12-mm nodules, and (**c**) a Bland–Altman plot showing the RVD and the LOA between the two systems for solid nodules. Notice the two clusters of measurements obtained by the DL CAD in **b**. RVD = relative volume difference, LOA = limit of agreement

**Fig. 5:**
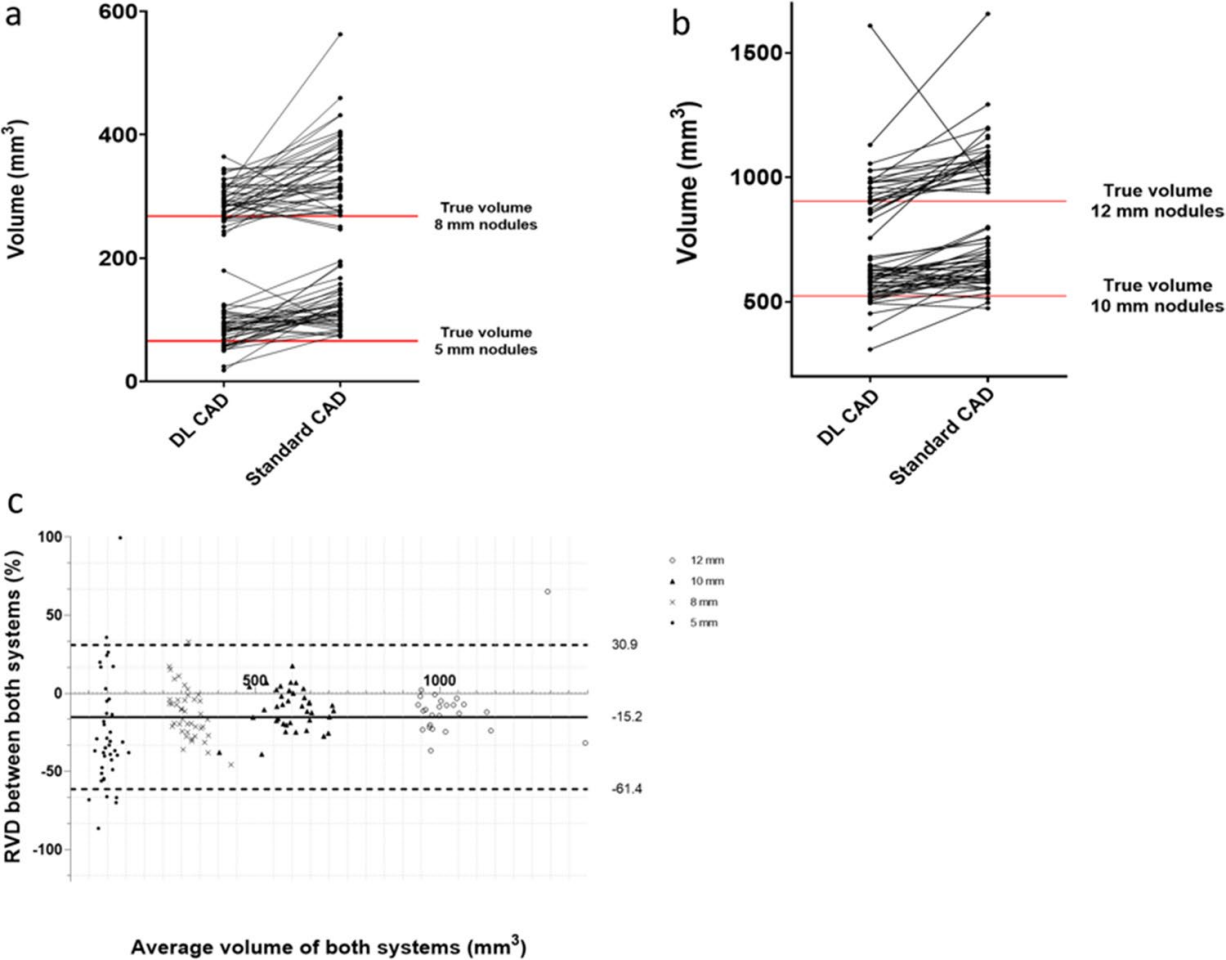
Software comparison depicted by spaghetti plots showing the dependent measurements of (**a**) 5-mm and 8-mm nodules and (**b**) 10-mm and 12-mm nodules and (**c**) a Bland–Altman plot showing the RVD and the LOA between the two systems for GGN. RVD = relative volume difference, LOA = limit of agreement

Three hundred nineteen nodules (174 solid, 145 GGN) were analyzed by both quantification systems. The DL CAD system showed higher measurement variability regarding the solid nodules, but less variability regarding the GGN, compared to the standard CAD system. The dependent measurements of both systems are depicted as spaghetti plots in Figs. 4 and 5.

As a coincidental finding, a clustering of the 10-mm and, even more pronounced, the 12-mm solid nodules was observed for the DL CAD measurements indicating a systematic error. Hereby, one nodule cluster was measured accurately; the second cluster was underestimated in size. Further workup of this observation revealed no correlation with nodule location or the maximum number of nodules per phantom (see Fig. 4b).

The Bland–Altman method was utilized to depict the RVDs between the systems. The resulting mean RVDs (SD) were 1.3 (18.6)% for solid nodules and − 15.2 (23.5)% for GGN with respective lower/upper limits of agreement (LOA) of − 35.2/37.7% and − 61.4/30.9% (Figs. 4c and 5c).

The sub-analyses of the 5-mm-, 8-mm-, 10-mm-, and 12-mm-size groups revealed that the RVDs were higher for the 5-mm nodules and GGN compared to those of the other groups. The RVDs and LOAs for all size groups and densities are shown in Table 2.

**Table 2:** RVD and LOA by size group and density

Nodule density	Size group (volume)	Mean RVD (SD), %	Lower/upper LOA, %
Solid	5 mm (65.5 mm^3^)	10.2 (23.6)	− 36.1/56.5
	8 mm (268.1 mm^3^)	5.0 (12.7)	− 19.9/29.9
	10 mm (523.6 mm^3^)	− 4.5 (11.3)	− 26.6/17.6
	12 mm (904.8 mm^3^)	− 11.4 (14.8)	− 40.4/17.6
GGN	5 mm (65.5 mm^3^)	− 25.6 (35.0)	− 94.4/43.1
	8 mm (268.1 mm^3^)	− 12.8 (16.0)	− 44.1/18.6
	10 mm (523.6 mm^3^)	− 10.2 (12.6)	− 34.9/14.5
	12 mm (904.8 mm^3^)	− 10.4 (18.1)	− 46.1/25.1

RVD and LOA of both CAD systems by size group and density

*GGN* ground-glass nodule, *LOA* limit of agreement, *RVD* relative volume difference, *SD* standard deviation

### Volumetric inaccuracies can have an impact on patient management

In order to evaluate the impact of the measurement differences on patient management, the hypothetical LungRADS categories of the solid nodules were compared between the systems. The DL-based CAD system classified 88.5% (*n* = 154/174) of all detected solid nodules correctly; the respective value for the standard CAD system was 79.8% (*n* = 142/178). The difference between the two systems was statistically significant (*p* = 0.004). The majority of the falsely classified nodules belonged to the 8-mm-size group; only one falsely classified nodule of each system measured 10 mm.

14.9% of the solid nodules (*n* = 26/174) were classified differently between the two systems (*p* = 0.002, Table 3), most of which were from the 8-mm-diameter group (*n* = 24) and the remaining two nodules from the 10-mm-diameter group. The 5-mm and 12-mm nodules would have all been classified in unison.

**Table 3:** LungRADS classification of solid nodules

		**Standard CAD**			
		**2**	**3**	**4A**	**Total**
**DL CAD**	**2**	52	0	0	52
	**3**	1	14	5	20
	**4A**	0	20	82	102
	**Total**	53	34	87	174

LungRADS classification of solid nodules by both systems

All GGN would have been categorized correctly as LungRADS 2 by both systems since no measurement exceeded the border to the LungRADS 3 classification (diameter, 30 mm; volume, 14'137.2 mm^3^).

## Discussion

The results of this work showed that changing the CAD system has a potential effect on the correct pulmonary nodule classification and therefore on patient management. Hereby, the DL-based CAD had a higher initial detection rate and classified a higher proportion of solid nodules correctly compared to the standard CAD system.

Inaccurate volumetry may lead to wrong lesion management decisions, which can either delay the correct diagnosis and treatment on the one side or cause unnecessary costs on the other, especially in the context of major lung cancer screening programs. Regarding patient management, changes in lesion size and the resulting potential shift in LungRADS categorization are critical. The DL-based CAD classified 88.5% of all solid nodules correctly according to LungRADS, which was significantly higher compared to the standard CAD system (79.8%, *p* = 0.004). Between the systems, 14.9% of the solid nodules were classified differently (*p* = 0.002). These numbers have to be interpreted with caution since most of the falsely classified nodules belonged to the 8-mm-size group, which is located right at the border between LungRADS categories 3 and 4A, implying that only minor volumetric mistakes can already lead to different classifications [24]. However, the experiment indicated that patient management can be affected by changing the CAD system.

The descriptive analysis indicated no clear difference between the three tube voltage groups in the current setting. It has to be mentioned that the DL-based software did not detect 19 nodules in the images acquired with 80 kV and therefore excluded them from the analysis, which most probably explains the observed difference between 80 and 120 kV regarding the GGN.

In contrast to the current observations, a previous study indicated that the alterations of tube current and voltage have an effect on the performance of this specific DL-based CAD system in the context of pulmonary nodule detection [25]. However, the current findings are in line with various other studies, which found CAD volumetry to be robust over a range of exposure settings [14].

The comparison of the density groups revealed that the DL-based CAD system was more precise in volumetry of the GGN and less precise in measuring solid nodules than the standard CAD. Standard CAD systems classically struggle with the detection of ground-glass lesions, due to the smaller density differences between normal lung and lesion [26–28]. The DL-based CAD system, on the other hand, was trained on both types, solid and subsolid lesions, which is reflected by the current results and is in line with their reported superiority over standard CAD systems in this regard [29].

In the analysis of the different size groups, the DL-based CAD system showed two measurement clusters regarding mainly the solid 12-mm nodules, indicating a systematic measurement error. This finding was most probably caused by the calibration dataset, which the software was trained on before the implementation; this dataset mainly contained small pulmonary nodules < 6 mm and may have led to a systematic underestimation of the larger nodule groups.

Apart from the solid 12-mm nodule group, both systems had the highest RVEs measuring the 5-mm nodules. This finding was somewhat to be expected, since only small measurement deviations can lead to high RVEs in this size group. Additionally, in this specific study, all nodules had been attached to vessels during the arrangement of the phantoms, making an accurate segmentation of the borders even more difficult. Correct segmentation of the nodule borders is crucial, as one pixel increase in this area may already alter the measured volume considerably [17]. In the case of the 5-mm GGN, the volumetric inaccuracy of the standard CAD system could even lead to the false assumption of nodule growth, since there was a volumetric error of > 25% [5, 30]

The mean RVD between the systems was 1.3 ± 18.6% for the solid nodules and – 15.2 ± 23.5% for the GGN. Bartlett and colleagues reported a mean RVD of − 0.9 ± 16.3% while assessing the interscan variability of 100 nodules measured twice with a standard CAD system [23]. For nodules < 80 mm^3^, they reported a mean RVD of − 0.3 ± 8.4%, which is much lower than the results for the respective group observed in the current study (10.2 ± 23.6% and – 25.6 ± 35.0% for solid nodules and GGN, respectively). A possible explanation is that Bartlett et al excluded all nodules with vascular or pleural attachment, which are more difficult to measure.

This study has several limitations. First, the protocol used is not state of the art for lung cancer screening according to the current guidelines by the European Society of Thoracic Imaging (ESTI), in particular referring to FBP as a reconstruction algorithm and the absence of overlapping image reconstruction [31]. However, the latest ACR guidelines are less strict as they only favor iterative reconstruction (IR) methods over FPB and do not recommend an overlapping reconstruction as mandatory [32]. Furthermore, it is a fact that many sites still use FBP for lung cancer screening [33], making the results of this study relevant for many institutions, especially the ones with only limited access to innovative scanner technologies [34]. In a previous phantom study, FBP and IR showed nearly identical results for pulmonary nodule volumetry using a software from the pre-AI era [14]. However, the same study setup is warranted for the AI era. Another important limiting aspect is the fact that the study was conducted on one single CT scanner with fixed settings; therefore, the results cannot be broadly transferred to other institutions or CT scanners. As already reported in various studies, the scanner settings such as reconstruction algorithm, slice thickness, etc. have a relevant impact on the performance of AI-based CAD systems [25, 35, 36]. In contrast to the current study, most of these studies were focused on pulmonary nodule detection rather than on volumetry. Another limitation is the use of artificial, perfectly spherical nodules with homogenous density and no surrounding pathology, which is of course not realistic and demands for repetition of this study in a clinical setting. However, the phantom setting of this study enabled a correlation with a perfect ground truth and an accurate evaluation of the CAD systems.

In conclusion, the DL-based system had a higher initial detection rate of pulmonary nodules and a higher proportion of them would have been classified correctly according to LungRADS compared to the standard CAD system. Nodule size and attenuation had an effect on the measurement accuracy of both systems; there was no effect of tube voltage. Our results indicate that measurement inaccuracies between CAD systems have a potential impact on patient management, which demands careful revision and, if needed, manual correction by radiologists.

## Abbreviations

ACR: American College of Radiology
AI: Artificial intelligence
AVD: Absolute volume difference
CAD: Computer-aided diagnosis
CT: Computed tomography
CTDIvol: Volume computed tomography dose index
DL: Deep learning
DLP: Dose length product
FBP: Filtered back projection
GGN: Ground-glass nodule
HU: Hounsfield unit
IR: Iterative reconstruction
LOA: Limit of agreement
Lung-RADS: Lung imaging reporting and data system
NELSON: Nederlands-Leuvens Longkaker Screening Onderzoek
NLST: National Lung Cancer Screening Trial
RVD: Relative volume difference
RVE: Relative volumetric error
Sv: Sievert

## Declarations

### Guarantor

The scientific guarantor of this publication is Dr. Peters.

### Statistics and biometry

One of the authors has significant statistical expertise (Moritz Pohl, MSc; Institute of Medical Biometry, University of Heidelberg, Heidelberg, Germany).

### Study subject or cohort overlap

The study subjects (chest phantoms) have been reported in a previous publication:

*Christe A, Szucs-Farkas Z, Huber A *et al *(2013) Optimal dose levels in screening chest CT for unimpaired detection and volumetry of lung nodules, with and without computer assisted detection at minimal patient radiation. PLoS One 8:e82919.*

In contrast to the current work, the study by Christe et al did not compare different CAD systems and furthermore did not involve deep-learning-based systems.

### Methodology

•Phantom study

•diagnostic or prognostic study

•performed at one institution
